# Portal Vein Thrombosis in Pediatric Liver Transplantation With Technical Variant Grafts: An International Multicenter Analysis of Risk and Prognostic Factors

**DOI:** 10.1097/TP.0000000000005777

**Published:** 2026-06-09

**Authors:** Kaihang Wang, Omid Madadi-Sanjani, Yuan Liu, Dimitri A. Raptis, Vasanthakumar Gunasekaran, Ashwin Rammohan, Seisuke Sakamoto, Hajime Uchida, Piotr Kaliciński, Nicolas Goldaracena, Cornelius J. van Beekum, Felix J. Krendl, Itsuko Chih-Yi Chen, Bryanna N. Domenick, Alfred Kow, Yuji Soejima, Katryn N. Furuya, George V. Mazariegos, Vidyadhar Padmakar Mali, Stephen Dunn, Kim Olthoff, Elizabeth B. Rand, Ian Leipnitz, Chao-Long Chen, Deniz Balci, Elvan Onur Kirimker, Silvio Nadalin, Stefan Schneeberger, Debra L. Sudan, Moritz Schmelzle, Uta Herden, Lutz Fischer, Blayne A. Sayed, Vicky L. Ng, Grzegorz Kowalewski, Mureo Kasahara, Mohamed Rela, Dieter C. Broering, Qiang Xia, Pierre-Alain Clavien, Zhihao Li

**Affiliations:** 1 University of Zurich, Zurich, Switzerland.; 2 Department of Visceral Transplantation, University Medical Center Hamburg-Eppendorf, Hamburg, Germany.; 3 Department of Liver Surgery, Renji Hospital Affiliated to Shanghai Jiao Tong University School of Medicine, Shanghai, China.; 4 Organ Transplant Center of Excellence, King Faisal Specialist Hospital and Research Center, Riyadh, Saudi Arabia.; 5 The Institute of Liver Disease and Transplantation, Dr Rela Institute and Medical Center, Chennai, India.; 6 Organ Transplantation Center, National Center for Child Health and Development (NCCHD), Tokyo, Japan.; 7 Department of Pediatric Surgery and Organ Transplantation, The Children’s Memorial Health Institute, Warsaw, Poland.; 8 Division of Abdominal Transplant Surgery, Department of Surgery, University Health Network, Toronto, ON, Canada.; 9 Department of General, Visceral and Transplant Surgery, Hannover Medical School, Hannover, Germany.; 10 Department of Visceral, Transplant and Thoracic Surgery, Center for Operative Medicine, Medical University of Innsbruck, Innsbruck, Austria.; 11 Department of Surgery, Kaohsiung Chang Gung Memorial Hospital, Kaohsiung, Taiwan.; 12 Division of Gastroenterology, Hepatology and Nutrition, Roberts Center for Pediatric Research, Children’s Hospital of Philadelphia, Philadelphia, PA.; 13 Division of Hepatobiliary and Pancreatic Surgery, Department of Surgery, National University of Singapore, Singapore, Singapore.; 14 Division of Gastroenterological, Hepato-Biliary-Pancreatic, Transplantation and Pediatric Surgery, Department of Surgery, Shinshu University School of Medicine, Matsumoto, Japan.; 15 Department of Pediatrics, University of Wisconsin-Madison School of Medicine and Public Health, Madison, WI.; 16 Hillman Center for Pediatric Transplantation, UPMC Children’s Hospital of Pittsburgh, Pittsburgh, PA.; 17 Department of Paediatric Surgery, National University Hospital, Singapore, Singapore.; 18 Department of Solid Organ Transplantation, Alfred I. duPont Hospital for Children, Wilmington, DE.; 19 Department of Surgery, Penn Transplant Institute, Perelman School of Medicine, University of Pennsylvania, Philadelphia, PA.; 20 Liver Transplant Unit, University of Auckland, Auckland, New Zealand.; 21 Department of General Surgery, Bahçesehir University Faculty of Medicine, İstanbul, Turkey.; 22 Department of General Surgery, Ankara University Faculty of Medicine, Ankara, Turkey.; 23 Department of General Visceral and Transplant Surgery, Eberhard Karls University of Tübingen, Tübingen, Germany.; 24 Division of Abdominal Transplantation, Department of Surgery, Duke University Medical Center, Durham, NC.; 25 Division of General & Thoracic Surgery, The Hospital for Sick Children, Toronto, ON, Canada.; 26 Wyss Zurich Translational Center, ETH Zurich and University of Zurich, Zurich, Switzerland.; 27 Department of Surgery and Transplantation, University of Zurich, Zurich, Switzerland.; 28 Division of Transplant Surgery, William J. von Liebig Transplant Center, Mayo Clinic, Rochester, Minnesota, MN.

## Abstract

**Background.:**

The adoption of technical variant grafts has expanded the donor pool for pediatric liver transplantation, but it also poses a risk of portal vein thrombosis (PVT). This study evaluated risk and prognostic factors of PVT in children undergoing liver transplantation with technical variant grafts.

**Methods.:**

A total of 3137 pediatric patients from 19 centers were included: 470 received split/reduced deceased donor grafts and 2667 received living donor grafts. Multivariable logistic regression was used to identify risk factors, whereas time-dependent Cox models were used to assess the impact on survival.

**Results.:**

PVT occurred in 102 patients (3.3%) and was associated with increased mortality (adjusted hazard ratio, 2.86; 95% confidence interval [CI], 1.13-7.25; *P* = 0.027) and graft loss (adjusted hazard ratio, 9.97; 95% CI, 4.90-20.3; *P* < 0.001). Risk factors included younger age, lower weight, higher Pediatric End-Stage Liver Disease score, longer cold ischemia and operation times, higher graft-to-recipient weight ratio, increased red blood cell transfusions, and biliary atresia. After adjusting for center, lower weight (odds ratio, 0.84 per kg; 95% CI, 0.71-1.00; *P* = 0.047) and biliary atresia (odds ratio, 2.34; 95% CI, 1.10-5.00; *P* = 0.027) remained predictors. Five-year patient (88.4% versus 93.1%) and graft survival (82.7% versus 96.3%) were significantly inferior in patients with PVT.

**Conclusions.:**

Low-weight children with biliary atresia constituted a high-risk group, highlighting the necessity for early detection and management.

## Graphical abstract

**Figure:**
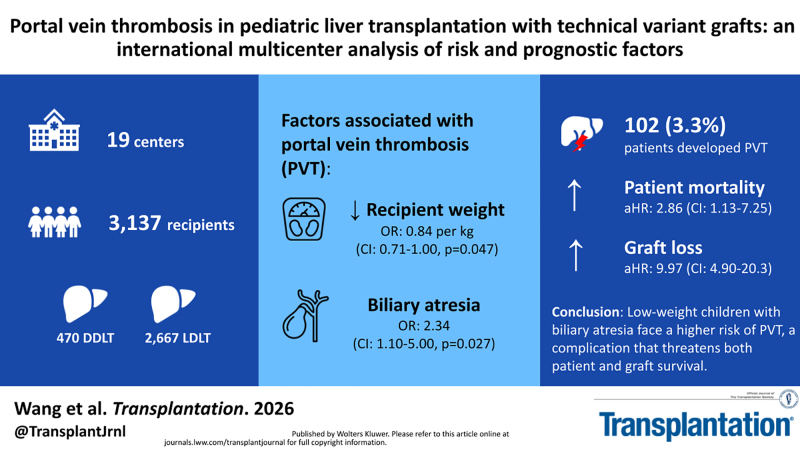

## INTRODUCTION

Pediatric liver transplantation (LT) is a rapidly progressing field that is performed at centers around the world. The evolution of surgical techniques, particularly the adoption of technical variant grafts (TVGs), such as living donor and split/reduced deceased donor grafts, has been an effective strategy in addressing organ shortage and reducing waitlist mortality.^1,2^ Although TVGs have been associated with superior outcomes in several studies, others have shown that vascular complications remain a chief cause of graft loss, particularly in children.^3-7^ Notably, portal vein thrombosis (PVT) has been reported to occur more frequently in TVGs compared with whole LTs.^8-10^ Furthermore, the reported incidence of 4%–16% in pediatric patients markedly exceeds that of adult recipients.^11-13^ This heightened risk is mainly due to the technical complexities of pediatric surgery, including the small caliber of the portal vein and significant size discrepancies between donor and recipient vessels.^14^ Clinically, PVT can range from asymptomatic to severe manifestations of portal hypertension, such as gastroesophageal variceal bleeding, ascites, and splenomegaly, potentially leading to graft failure.^6^ Prior research has identified potential risk factors for PVT, such as low recipient weight, a small portal vein, and the use of vascular grafts.^5,12,13,15,16^ However, the evidence is limited by single-center designs and small cohorts. Furthermore, the effect of PVT on long-term survival remains uncertain. Therefore, this large-scale, international multicenter study aims to identify risk factors for PVT after pediatric LT and evaluate the impact of PVT on graft and patient outcomes.

## MATERIALS AND METHODS

### Study Population

This international, multicenter, retrospective study included 3137 consecutive pediatric patients (younger than 18 y) who underwent LT with TVGs between January 1, 2019, and December 31, 2023. Data were contributed by 19 participating centers, 7 of which were high-volume (**Figure S1, SDC,** https://links.lww.com/TP/D406). Patients who underwent retransplantation or multiorgan transplantation were excluded. Ethical approval was granted by the Cantonal Ethics Commission of Zurich (BASEC 2024-01544) and by all participating centers, which waived the need for consent due to the study’s retrospective, low-risk design.

### Definition of Variables

PVT was diagnosed via routine Doppler ultrasonography, with cross-sectional imaging (CT or MRI) used to confirm equivocal cases. Based on the time from transplantation, PVT was classified as early (≤10 d) or late (>10 d).^14^ Management was classified as surgical (thrombectomy or anastomotic revision) or nonsurgical (anticoagulation therapy or radiological intervention). Radiological interventions included percutaneous transluminal angioplasty with or without stenting. Anticoagulation involved low-molecular-weight heparin, supplemented in some cases with aspirin. Portal venous flow was standardized by graft weight (mL/100 g/min), and low portal flow was defined as <150 mL/100 g/min.^13^ Portal modulation involved ligation of portosystemic shunts and collaterals, including the splenorenal, gastrorenal, left gastric, and inferior mesenteric veins, or splenectomy. Successful recanalization was defined as the restoration of portal venous flow, verified by Doppler ultrasound or cross-sectional imaging. Patient survival was defined as the time from transplantation to death or last follow-up, and graft survival as the time from transplantation to graft loss, censoring for death with a functional graft.

### Perioperative Practices

Given the multicenter nature of the study, perioperative management followed institutional standards. Among the 19 centers, 16 reported routine use of postoperative anticoagulation or antiplatelet therapy, whereas 3 centers used no routine prophylaxis. Most institutions use intravenous unfractionated heparin or low-molecular-weight heparin for the first 7–14 d posttransplant, often targeting an activated partial thromboplastin time of 30–60 s. Following the initial heparin phase, the transition to oral antiplatelet therapy (primarily aspirin) is standard, with durations ranging from 3 mo to 1 y.

All participating centers use a standardized, intensive Doppler surveillance protocol in the early postoperative period. Monitoring frequency typically ranges from 1 to 3 times daily during the first 3–7 postoperative days, subsequently transitioning to daily or twice-weekly scans until discharge. Clinical triggers for unscheduled Doppler studies were largely consistent and included rising liver enzymes, suspected bleeding or thrombosis, fever, unexplained coagulation changes, and increased ascites.

### Statistical Analysis

Continuous variables were summarized as medians with interquartile ranges and compared using the Mann-Whitney *U* test. Categorical data were expressed as counts with percentages and compared using the chi-square test.

#### Risk Factor Identification for PVT

Potential risk factors for PVT were initially evaluated using univariable logistic regression. To reduce overfitting, variable selection for multivariable modeling was performed among clinically relevant variables using Least Absolute Shrinkage and Selection Operator regression (**Table S1, SDC,** https://links.lww.com/TP/D406). The predictors identified by the Least Absolute Shrinkage and Selection Operator were then entered into a mixed-effects multivariable logistic regression model. This model treated the transplant center as a random intercept to account for center-level variation while estimating fixed effects for the clinical predictors. Findings from this analysis are reported as odds ratios (ORs) with 95% confidence intervals (CIs).

#### Survival Analysis

Patient and graft survival were estimated using the Kaplan-Meier method and compared between groups stratified by PVT status using the log-rank test. To adjust for potential confounders, multivariable Cox proportional hazards models were used to assess the independent effect of PVT on patient and graft survival, adjusting for recipient age, sex, weight, Pediatric End-Stage Liver Disease (PELD) score, and biliary atresia (BA). Results are presented as adjusted hazard ratios (aHRs) with 95% CIs. To minimize immortal time bias, a time-dependent covariate was incorporated into the Cox models. Patients were initially categorized as non-PVT and subsequently reclassified to the PVT group at the time of diagnosis. Outcomes such as graft loss or death were assigned according to PVT status at the time of the event, ensuring accurate estimation of the impact of PVT over time.

Given the particular clinical relevance of PVT in patients with BA, a subgroup analysis was performed for this population. Potential risk factors for PVT in patients with BA were evaluated using univariable logistic regression. For multivariable analysis, clinically relevant variables were entered into a Firth regression model, adjusting for Kasai portoenterostomy, portal modulation, portal vein reconstruction, weight, and age. The impact of PVT on mortality and graft loss in patients with BA was evaluated using (1) univariable analysis; (2) minimal adjustment for Kasai portoenterostomy status using Firth regression; and (3) full adjustment for age, sex, weight, and center effects using cluster-robust standard errors. Kaplan-Meier survival curves were generated to visualize differences in both patient and graft survival, with log-rank tests used to assess statistical significance.

#### Analysis of PVT Management and Outcomes

The interval from the date of PVT diagnosis to recanalization was calculated. The association between the timing of PVT and the treatment approach was analyzed. Finally, the impact of surgical and nonsurgical treatments on recanalization success was evaluated using cumulative incidence curves.

All analyses were performed using R version 4.5.1. (R Foundation for Statistical Computing, Vienna, Austria). A 2-tailed *P* value of <0.05 was considered statistically significant.

## RESULTS

### Study Population

**FIGURE 1.**
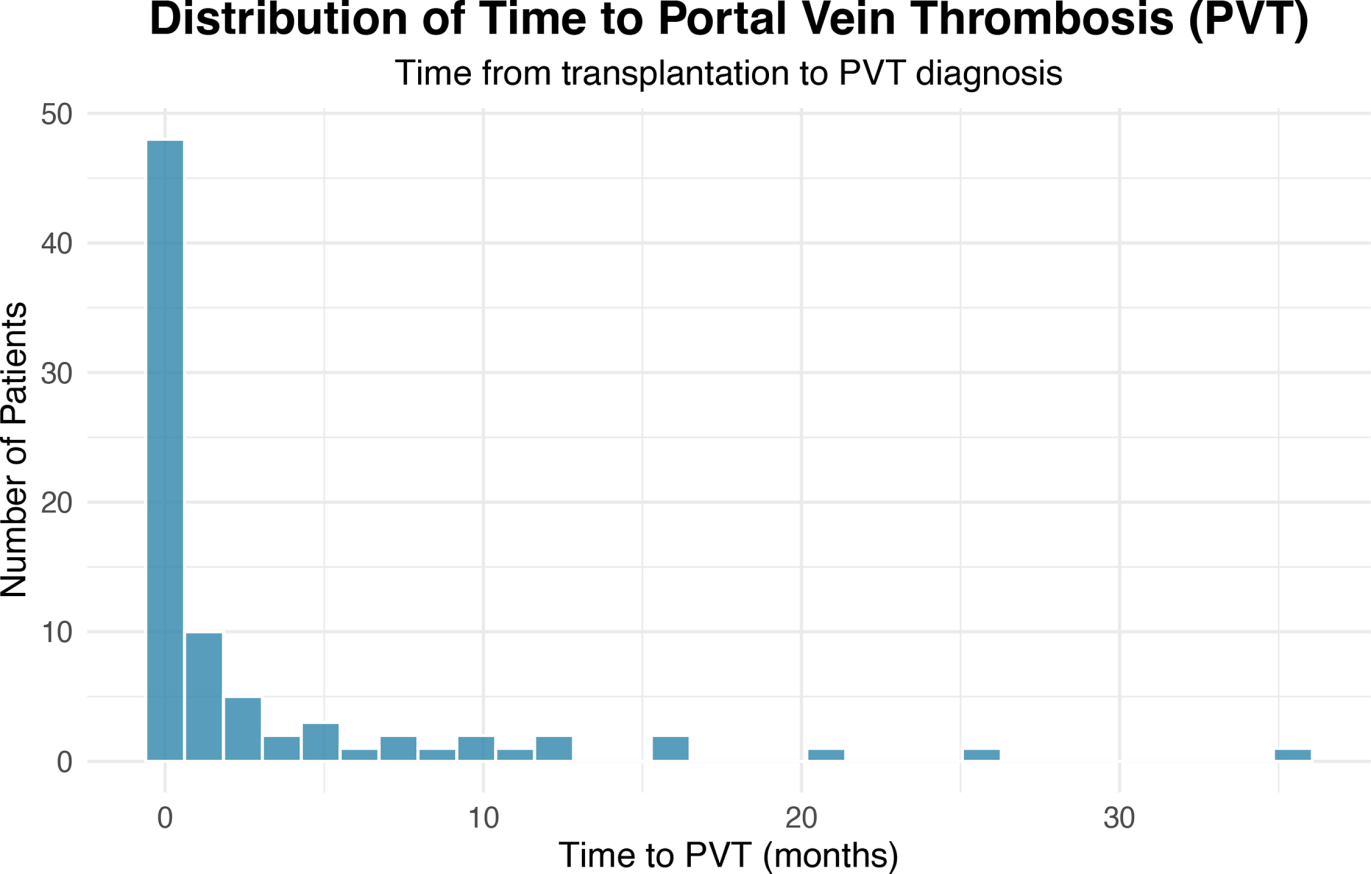
Distribution of time to PVT. PVT, portal vein thrombosis.

A total of 19 centers participated in the study, including 7 high-volume centers. The centers are distributed across 4 continents: Europe (5), Asia (8), North America (5), and Oceania (1). Across all centers, 3137 patients were included: 470 received split/reduced deceased donor grafts and 2667 underwent living donor LT (LDLT). PVT occurred in 102 patients, corresponding to an overall incidence of 3.3%. The median time to PVT diagnosis was 10 d (interquartile range, 1–86), with a right-skewed distribution showing the highest incidence within the first 3 mo (Figure 1). Categorically, 51% of cases were “early” (≤10 d) and 49% were “late” (>10 d). Baseline recipient, donor, and graft characteristics by PVT status are summarized in Table 1. Recipients who developed PVT were younger (median 8 versus 12 mo, *P* < 0.001) and had lower body weight (median 7 versus 8.5 kg, *P* < 0.001). The underlying liver condition differed between groups, with BA being notably more prevalent in the PVT group (79.4% versus 55.8%, *P* < 0.001). Consequently, a prior Kasai procedure was also more common in the PVT group (54.9% versus 42.8%, *P* = 0.006). Furthermore, the PVT group had a higher median PELD score (19 versus 16, *P* = 0.015), a higher graft-to-recipient weight ratio (GRWR; 3.5 versus 2.9, *P* = 0.001), and longer operation durations (400 versus 380 min, *P* = 0.011).

**TABLE 1.** Baseline characteristics of recipient, donor, and graft by the presence of PVT

Characteristics	No PVT (N = 3035)	PVT (N = 102)	*P*
Age, mo, median (IQR)	12 (6.5–39)	8 (6–12)	<0.001
Sex, male, n (%)	1532 (50.5%)	45 (44.1%)	0.227
Weight, kg, median (IQR)	8.5 (6.6–14)	7 (6–8.3)	<0.001
PVT pretransplant, n (%)	66 (2.2%)	4 (3.9%)	0.288
Cause of liver disease, n (%)			
Biliary atresia	1694 (55.8)	81 (79.4)	<0.001
Other cholestatic diseases	370 (12.2)	8 (7.8)	0.217
Metabolic diseases	503 (16.6)	3 (2.9)	<0.001
Pediatric acute liver failure	135 (4.4)	6 (5.9)	0.462
Tumor	139 (4.6)	1 (1.0)	0.088
Others	193 (6.4)	3 (2.9)	0.211
Kasai procedure, n (%)	1299 (42.8%)	56 (54.9%)	0.006
PELD score, median (IQR)	16 (6.4–24.2)	19 (12.7–25.2)	0.015
MELD score, median (IQR)	16 (9–25)	12 (9.3–24.5)	0.870
Graft weight, g, median (IQR)	260 (220–312)	250 (211–285)	0.021
GRWR, median (IQR)	2.9 (2–3.8)	3.5 (2.4–4.3)	0.001
Cold ischemia time, min, median (IQR)	43 (23–138)	84 (25–182)	0.031
Warm ischemia time, min, median (IQR)	30 (30–44)	36.5 (30–46.8)	0.027
Operation duration, min, median (IQR)	380 (318.5–448)	400 (319.3–509)	0.011
Preoperative bilirubin levels, mg/dL, median (IQR)	109 (16.8–246)	123.3 (35.2–263)	0.192
Preoperative platelet count, μL, median (IQR)	181 (107–290)	158 (85–228)	0.069
Intraoperative RBC transfusions, 100 mL, median (IQR)	2 (1–4)	3.2 (2–5.3)	<0.001
Portal vein flow after reperfusion, mL/100 g/min, median (IQR)	477 (339.7–689.4)	459.5 (240.3–718.3)	0.330
Graft type, n (%)			
Left lateral segment graft	2530 (85.8)	84 (89.4)	0.407
Left lobe graft	323 (11)	5 (5.3)	0.118
Right lobe graft	44 (1.5)	2 (2.1)	0.946
Extended right lobe graft	10 (0.3)	0 (0)	1.000
Donor type, n (%)			0.384
Deceased transplant	455 (15)	15 (14.7)	
Living donor transplant	2580 (85)	87 (85.3)	
Reduced graft	381 (14.6)	14 (15.4)	0.795

GRWR, graft-to-recipient weight ratio; IQR, interquartile range; MELD, Model for End-Stage Liver Disease; PELD, Pediatric End-Stage Liver Disease; PVT, portal vein thrombosis; RBC, red blood cell.

### Predictors of PVT

Upon univariable analysis, younger recipient age, lower recipient weight, the underlying diagnosis of BA or other cholestatic diseases, a prior Kasai procedure, higher PELD score, higher GRWR, and longer operation duration were associated with an increased risk of PVT (Table 2). The need for graft reduction and a portal venous flow <150 mL/100g/min were not identified as significant risk factors. Subsequent multivariable analysis identified younger recipient age, lower recipient weight, higher PELD score, longer cold ischemia time, longer operation duration, higher GRWR, greater red blood cell transfusion volume, and the diagnosis of BA as independent risk factors for PVT. After adjustment for center effects, BA remained a strong independent predictor of PVT (OR, 2.34; 95% CI, 1.10-5.00; *P* = 0.027), whereas higher recipient weight was protective (OR, 0.84 per kg; 95% CI, 0.71-1.00; *P* = 0.047; Table 3). The influence of ischemia times and operation duration was attenuated after accounting for center effects. Furthermore, after adjusting for donor source (living versus deceased) and graft type, cold and warm ischemia times were not independently associated with PVT (**Table S3, SDC,** https://links.lww.com/TP/D406).

**TABLE 2.** Results of univariable and multivariable logistic regression

Characteristics	OR (95% CI)	*P*	OR (95% CI)
Age, mo	0.99 (0.98-1.00)	0.006	0.95 (0.93-0.98)
Sex, male	0.77 (0.52-1.15)	0.206	
Weight, kg	0.96 (0.92-0.99)	0.007	0.95 (0.81-1.12)
PVT pretransplant	1.84 (0.55-4.55)	0.247	
Cause of liver disease			
Biliary atresia	3.05 (1.92-5.08)	<0.001	1.45 (0.68-3.07)
Other cholestatic diseases	3.22 (1.86-6.07)	<0.001	
Metabolic diseases	0.15 (0.04-0.41)	0.001	
Pediatric acute liver failure	1.34 (0.52-2.87)	0.494	
Tumor	0.21 (0.01-0.93)	0.117	
Kasai procedure	1.81 (1.20-2.75)	0.005	
PELD score	1.02 (1.00-1.04)	0.018	1.09 (1.07-1.12)
MELD score	0.99 (0.90-1.07)	0.813	
Graft weight, g	1.00 (0.99-1.00)	0.109	
GRWR	1.12 (1.02-1.22)	0.010	1.03 (0.80-1.32)
Reduced graft	0.94 (0.47-1.72)	0.86	
Cold ischemia time, h	1.04 (0.97-1.11)	0.220	1.08 (0.98-1.18)
Warm ischemia time, h	0.92 (0.80-1.02)	0.143	
Operation duration, h	1.15 (1.06-1.25)	<0.001	1.22 (1.10-1.36)
Preoperative bilirubin levels, mg/dL	1.00 (1.00-1.00)	0.527	
Preoperative platelet count, μL	1.00 (1.00-1.00)	0.104	
Intraoperative RBC transfusions (100 mL)	1.02 (0.99-1.04)	0.074	1.05 (1.00-1.11)
Portal vein flow after reperfusion <150 mL/min/100 g	0.90 (0.45–1.71)	0.749	
Portal modulation	3.35 (1.78-5.91)	<0.001	
ABO-incompatibility	1.32 (0.74-2.23)	0.315	

CI, confidence interval; GRWR, graft-to-recipient weight ratio; MELD, Model for End-Stage Liver Disease; OR, odds ratio; PELD, Pediatric End-Stage Liver Disease; PVT, portal vein thrombosis; RBC, red blood cell.

**TABLE 3.** Results of multivariable analysis after adjustment for center effects

Characteristics	OR (95% CI)	*P*
Age, mo	1.01 (0.98-1.04)	0.393
Weight, kg	0.84 (0.71-1.00)	0.047
PELD score	1.02 (0.99-1.04)	0.147
Cold ischemia time, h	0.98 (0.86-1.12)	0.807
Operation duration, h	1.05 (0.93-1.20)	0.425
GRWR	0.86 (0.66-1.13)	0.275
Intraoperative RBC transfusions (100 mL)	1.03 (0.97-1.10)	0.287
Biliary atresia	2.34 (1.10-5.00)	0.027

CI, confidence interval; GRWR, graft-to-recipient weight ratio; OR, odds ratio; PELD, Pediatric End-Stage Liver Disease; RBC, red blood cell.

### Impact on Survival and Management Strategies

**FIGURE 2.**
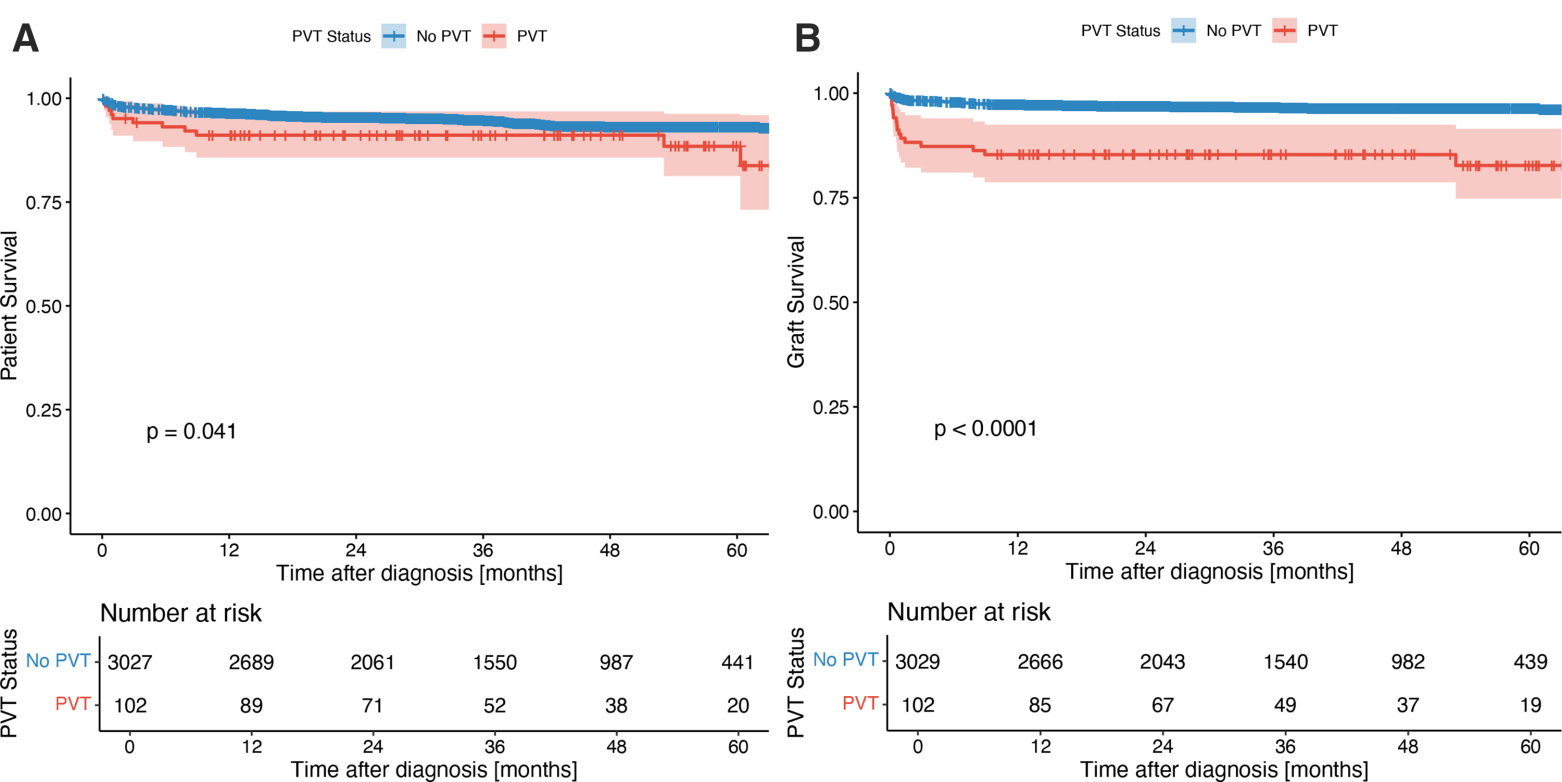
Comparison of patient survival (A) and graft survival (B) by presence of PVT (log-rank test). PVT, portal vein thrombosis.

The occurrence of PVT was associated with significantly worse patient (aHR, 2.86; 95% CI, 1.13-7.25; *P* = 0.027) and graft survival (aHR, 9.97; 95% CI, 4.90-20.3; *P* < 0.001; Table 4). Patients without PVT showed 1-, 3-, and 5-y patient survival of 96.4%, 94.7%, and 93.1%, respectively (Figure 2). By contrast, patients with PVT had worse survival at each time point: 91.1% at 1 y, 91.1% at 3 y, and 88.4% at 5 y. The negative impact of PVT was even greater for graft survival. In non-PVT patients, survival rates were 97.2%, 96.6%, and 96.3% at 1, 3, and 5 y, whereas grafts in patients with PVT had significantly lower survival rates of 85.3%, 85.3%, and 82.7% at the same intervals.

**TABLE 4.** Impact of portal vein thrombosis on graft and patient survival

	Patient survival		Graft survival	
Prognostic factors	HR (95% CI)	*P*	HR (95% CI)	*P*
Portal vein thrombosis	2.86 (1.13-7.25)	0.027	9.97 (4.90-20.3)	<0.001
Age, mo	1.00 (0.99-1.01)	0.5	0.99 (0.99-1.00)	0.11
Male sex	1.13 (0.81-1.59)	0.5	1.17 (0.79-1.73)	0.4
Weight, kg	1.00 (0.98-1.03)	0.7	1.02 (1.00-1.05)	0.060
PELD	1.01 (1.00-1.03)	0.11	1.02 (1.00-1.04)	0.019
Biliary atresia	0.57 (0.46-0.71)	<0.001	0.34 (0.17-0.67)	0.002

CI, confidence interval; HR, hazard ratio; GRWR, graft-to-recipient weight ratio; PELD, Pediatric End-Stage Liver Disease; RBC, red blood cell.

**FIGURE 3.**
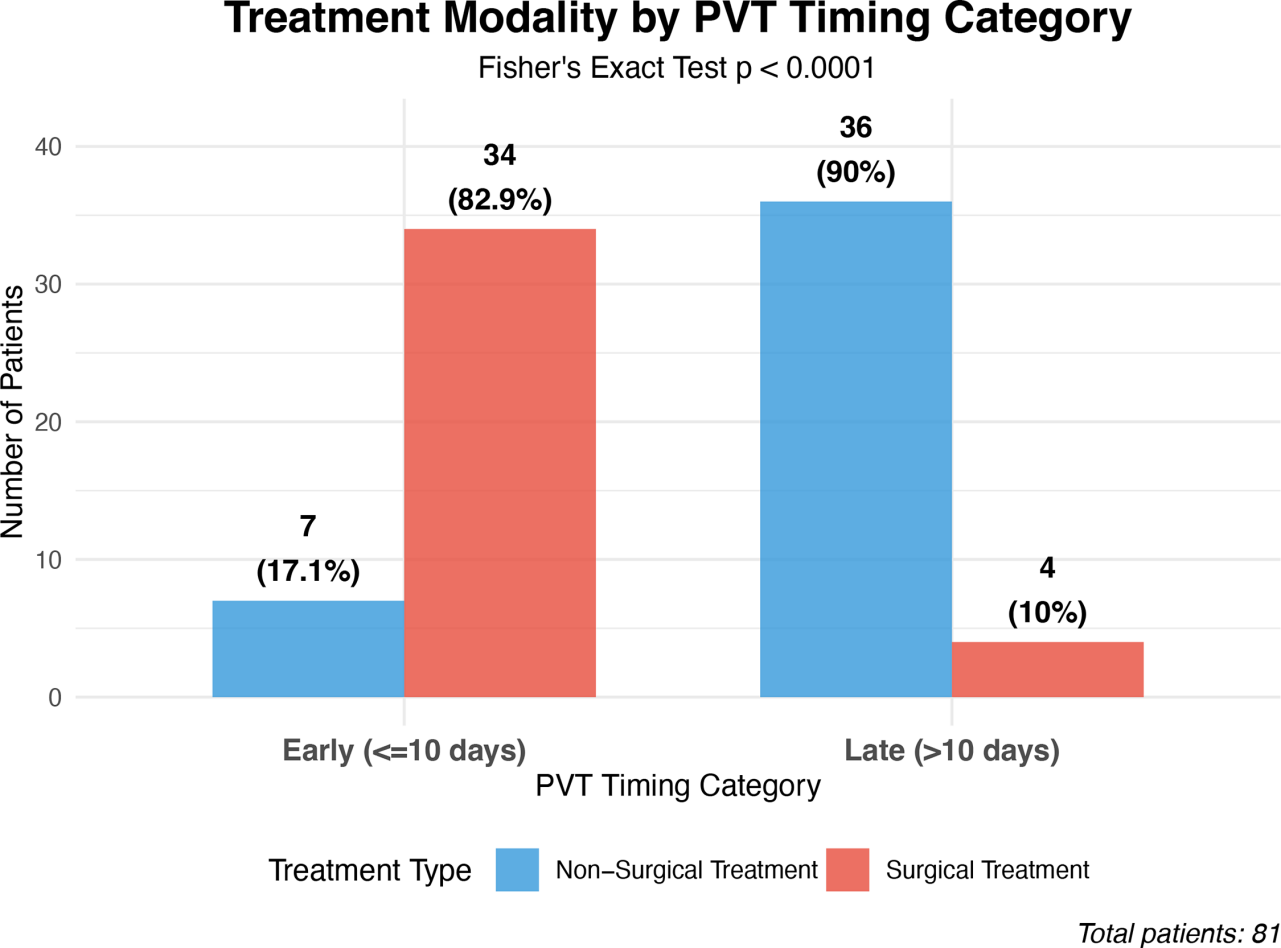
Treatment modality by PVT timing category (Fisher exact test). PVT, portal vein thrombosis.

**FIGURE 4.**
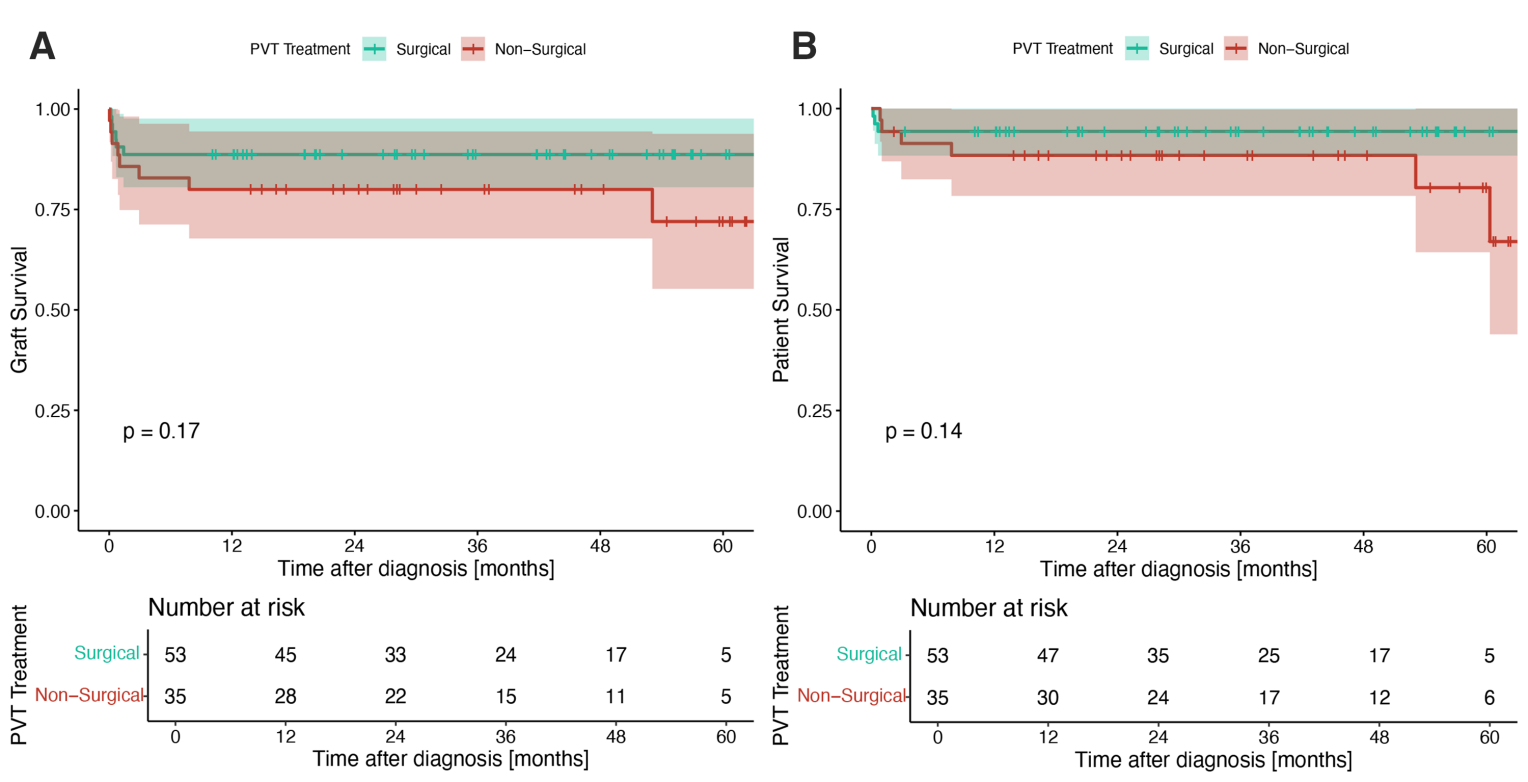
Comparison of patient survival (A) and graft survival (B) following surgical and nonsurgical management of PVT (log-rank test). PVT, portal vein thrombosis.

Among the 87 patients with available treatment data, 43 underwent surgical management and 44 received nonsurgical management. Patients in the surgical group had longer cold ischemia times (median 120 versus 25 min, *P* = 0.001), warm ischemia times (median 39 versus 30 min, *P* < 0.001), and operation durations (median 7.48 versus 6.15 h, *P* = 0.004). As expected, anticoagulation was more frequently used in the nonsurgical group (77.3% versus 44.2%, *P* = 0.003; **Table S2, SDC,** https://links.lww.com/TP/D406). The choice of treatment modality was associated with the timing of PVT diagnosis (*P* < 0.001): surgical treatment was more common in early PVT (83%), whereas nonsurgical treatment was the predominant approach in late PVT (90%; Figure 3). The overall cumulative incidence of recanalization reached a plateau of 92% by 64 d postdiagnosis. Surgical management achieved a 100% recanalization rate (21/21 patients) within 60 d (**Figure S2, SDC,** https://links.lww.com/TP/D406). In addition, a comparative analysis for nonsurgical management was not possible due to insufficient follow-up data. Patients treated surgically had 1-, 3-, and 5-y patient survival of 94.3%, 94.3%, and 94.3%, respectively. The nonsurgical group had patient survival of 88.4%, 88.4%, and 80.4% at the same intervals. However, no significant difference in patient or graft survival was observed between the 2 treatment groups (Figure 4).

### Subgroup Analysis in BA

**FIGURE 5.**
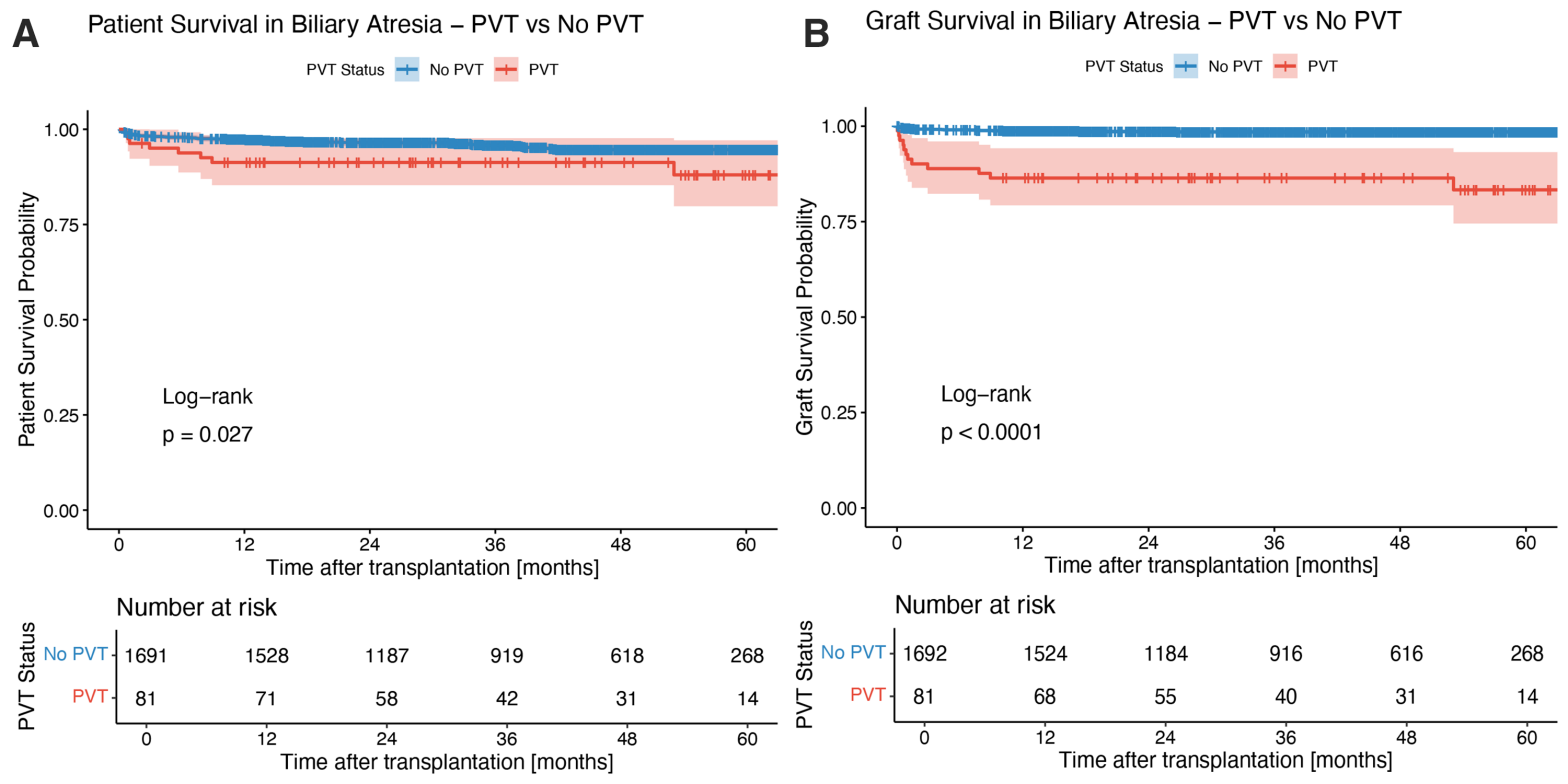
Comparison of survival outcomes by presence of PVT among biliary atresia patients (log-rank test). PVT, portal vein thrombosis.

Given that BA was the most prevalent etiology (n = 1775) and a strong independent predictor of PVT in the overall cohort, a subgroup analysis was performed on this population (**Table S4, SDC,** https://links.lww.com/TP/D406). The recipients who developed PVT (n = 81) were similarly young (median 7.1 versus 8 mo, *P* = 0.085) but had a lower body weight (median 7 versus 7.3 kg, *P* = 0.004) than those without PVT. The higher PELD score observed in the overall cohort was not observed (median 18.0 versus 17.3, *P* = 0.290), and the association with a prior Kasai procedure was no longer present (73% versus 77.8%, *P* = 0.412; **Table S4, SDC,** https://links.lww.com/TP/D406). Portal modulation (OR, 3.29; 95% CI, 1.76-6.17; *P* = 0.001) and portal vein reconstruction (OR, 2.96; 95% CI, 1.7-5.14; *P* < 0.001) were independent risk factors for developing PVT, whereas prior Kasai procedure, recipient age, and lower weight were not independently associated with PVT risk (**Table S5, SDC,** https://links.lww.com/TP/D406). In adjusted Cox models, PVT was an independent predictor of both patient mortality (aHR, 2.2; 95% CI, 0.86-5.62; *P* = 0.10) and graft loss (aHR, 11.86; 95% CI, 6.07-23.1; *P* < 0.001), even after adjusting for Kasai status and accounting for center-level effects (**Table S6, SDC,** https://links.lww.com/TP/D406). Consistent with the outcomes of the overall cohort, non-PVT patients demonstrated better outcomes in both patient survival and graft survival. 1-, 3-, and 5-y patient survival was 97.3%, 95.8%, and 94.7% for patients without PVT, and 91.3%, 91.3%, and 88% for patients with PVT (Figure 5). Graft survival rates were 98.7%, 98.5%, and 98.5% at 1, 3, and 5 y, whereas grafts in patients with PVT had significantly lower survival rates of 86.4%, 86.4%, and 83.3% at the same intervals.

### Subgroup Analysis in Smaller Children

As low recipient weight was a strong independent predictor of PVT in the general cohort, a separate analysis was performed in children weighing <10 kg and younger than 5 y (**Table S7, SDC,** https://links.lww.com/TP/D406). Within this cohort, PVT was associated with a 10-fold increase in the risk of graft loss (aHR, 10.0; 95% CI, 4.87-20.6; *P* < 0.001). Furthermore, PVT showed a strong trend toward reduced patient survival, although it did not reach statistical significance (aHR, 2.61; 95% CI, 0.99-6.85; *P* = 0.052). Multivariable analysis showed that the use of portal flow modulation (OR, 4.17; 95% CI, 1.86-9.35; *P* = 0.001) and portal vein reconstruction (OR, 2.11; 95% CI, 1.11-4.01; *P* = 0.023) were independently associated with the development of PVT. Notably, factors that were significant in the general population, such as the diagnosis of BA, did not reach statistical significance in this subgroup.

## DISCUSSION

This multicenter study provides an analysis of the risk factors and impact of PVT following pediatric LT with TVGs. Not only is LDLT increasingly used for pediatric LT to overcome organ shortage, but it has also been associated with superior patient survival.^3,4^ However, these procedures are technically demanding and carry a higher risk of PVT compared with whole LT.^8-10^

The overall incidence of PVT in our cohort (3.3%) falls at the lower end of the previously reported range of 4%–16% in pediatric LT.^11-13^ This likely reflects the greater surgical expertise and potentially more selective patient inclusion of the participating high-volume centers.

Low body weight is often associated with smaller vascular caliber, which may complicate the anastomosis and promote flow disturbances that predispose to thrombosis.^17^ Cheng et al^5^ found that infants younger than 1 y often have portal vein diameters <4 mm, which considerably increased the risk for thrombosis.^18^ Furthermore, Ueda et al^19^ found that bodyweight <6 kg was the only risk factor for PVT among 521 pediatric LDLTs. As low-weight recipients typically have smaller abdominal cavities, graft compression and kinking of the portal vein after closure would increase intrahepatic vascular resistance and reduce portal inflow.^11^ These mechanisms might explain the predominance of early PVT observed in this study and others.^11,15,16^ Thus, the use of smaller or monosegmental grafts and delaying abdominal wall closure in small recipients have been recommended to mitigate compression-related thrombosis.^20^ In our analysis, the requirement for technical graft reduction was not identified as a significant predictor of PVT. This suggests that although these modifications are often required by the challenging anatomy of very small recipients to avoid “large-for-size” complications, they do not independently increase the risk of PVT. Furthermore, although GRWR and recipient age were not independently predictive after multivariable adjustment, the close clinical association between young age and low weight remains important.

Prolonged cold ischemia time can exacerbate endothelial injury, whereas increased red blood cell transfusion likely reflects intraoperative hemorrhage, both of which can promote thrombosis.^21-23^ The reduced effects after adjustment for center may be explained by differences among institutions, such as surgical technique and donor-related protocols. When adjusting for donor source and graft type, ischemia times did not remain independent risk factors, implying that their impact is likely mediated by these surgical and institutional factors. Nevertheless, these intraoperative factors remain clinically relevant indicators of operative stress.

Other studies have similarly discovered that patients with BA have a higher risk for posttransplant vascular complications.^5,14,15,17^ BA is characterized by inflammation and fibrosis, which induces early portal hypertension. The resulting shunting of mesenteric blood away from the liver leads to reduced portal flow and hypoplasia of the portal vein, promoting PVT.^14,24^ To manage these challenges, interposition grafts are used for reconstruction, and portal flow modulation—in our case, the ligation of collateral shunts to mitigate steal syndrome—is frequently required.^14,25^ In our analysis, these technical interventions, rather than recipient age or weight, emerged as the only independent predictors of PVT within the BA subgroup. This suggests that the risk of PVT is driven by the severity of portal hypoplasia and the complexity of the surgical correction required to achieve adequate flow, rather than the recipient size alone. Furthermore, although the scarring from a prior Kasai portoenterostomy adds technical difficulty and its impact on outcomes remains debated,^26^ our data suggest that the preoperative condition of the portal system itself determines the risk of thrombosis.

In children younger than 5 y and weighing <10 kg, lower age and weight were no longer predictors of PVT risk, likely reflecting the reduced variance within this subgroup. Due to the high prevalence of BA, the diagnosis itself was not a significant predictor, whereas portal flow modulation and portal vein reconstruction remained associated with PVT risk. PVT persists as the most significant threat to graft survival among these small recipients. However, it was no longer an independent predictor of patient mortality, suggesting that its occurrence does not absolutely determine patient survival in this vulnerable population.

While studies report outcomes ranging from no effect to worse survival,^11,12,17,19^ our analysis found that PVT was positively associated with patient mortality and graft loss. In contrast, a diagnosis of BA was associated with better survival outcomes compared with other indications, such as pediatric acute liver failure, metabolic diseases, and other cholestatic disorders. Likewise, Sanada et al^27^ reported superior 10-y patient and graft survival in a cohort of 302 LDLT recipients with BA. For patients with BA, LT is a definitive, curative procedure. In comparison, patients with inherited metabolic diseases may have persistent extrahepatic manifestations of their underlying disorder, and those with acute liver failure often have a poorer preoperative condition and face the risk of disease recurrence, all of which can negatively impact long-term outcomes.^27,28^

Finally, management of PVT is crucial but lacks consensus, due to limited evidence and varying preferences and expertise among centers. Early thromboses are often acute complications requiring immediate surgical flow restoration, whereas late PVTs tend to be more insidious and hemodynamically stable, allowing for less invasive approaches.^11,19,29,30^ Ueda et al^19^ reported mortality in all patients with early PVT (≤3 mo) unless portal flow was surgically restored, implying that surgical treatment, such as thrombectomy, is essential for survival. Similarly, Karakas et al^30^ and Corno et al^29^ associated early PVT (≤10 d) with increased mortality but with potential reversibility through surgical revision. In our study, surgical management achieved high recanalization rates. However, this result should be interpreted with caution, as this finding is based on a limited number of patients (n = 21). Patient and graft survival did not differ significantly between surgical and nonsurgical treatments, suggesting that timely detection and individualized management may be more important than the specific therapeutic approach.

### Limitations

We are aware that this study has several limitations. First, its retrospective, multicenter design introduces inherent biases related to data collection and reporting. Although the international scope provided a large, diverse study population, it also introduced variability in diagnostic criteria, treatment approaches, and surgical techniques across centers. Although most centers use routine anticoagulation, the specific agents and duration vary widely. Similarly, postoperative Doppler surveillance protocols differed in intensity, which may influence the reported incidence of PVT and the timing of its detection. Second, case volume varied across participating centers, with high-volume centers contributing the majority of cases. Such an imbalance may bias findings toward the practices and outcomes of larger centers. However, we used a mixed-effects model with center as a random intercept to account for this clustering. Although these methods strengthen the internal validity of our risk factor analysis, we acknowledge that they cannot entirely eliminate residual confounding from unmeasured factors such as institutional experience or specific local management protocols. Third, other potential risk factors for PVT, such as preoperative PVT, type of interposition graft, and intraoperative portal flow measurements, were not uniformly documented across all participating centers and therefore could not be analyzed. Furthermore, anatomical data of the portal vein, specifically portal vein size, hypoplasia, and reconstruction techniques, were unavailable or inconsistently reported. Although the need for portal flow modulation or portal vein reconstruction serves as a surrogate for technically demanding anatomy, particularly in the BA population, it is ultimately the underlying anatomy that determines patient outcome. Despite interventions, the risk of PVT remains relevant. Therefore, significant progress is still needed in developing strategies to effectively minimize this risk. Finally, it was not possible to investigate the treatment efficacy further due to the lack of consistent follow-up data. Consequently, our comparison between surgical and nonsurgical management of PVT is limited by a small sample size and potential selection bias, as treatment decisions and outcomes were likely influenced by individual patient characteristics and institutional expertise. Further data collection would be required to make comparisons of recanalization rates between surgical and nonsurgical treatment modalities.

## CONCLUSIONS

PVT is a serious complication that threatens patient and graft survival. However, our data demonstrate that excellent outcomes are achievable with close surveillance and timely treatment. Low body weight and a diagnosis of BA are significant risk factors for PVT. These findings emphasize the clinical importance of identifying high-risk patients, particularly low-weight infants with BA. Ultimately, measures to improve early detection and management should be researched in future studies to mitigate the impact of this complication. We hope that our results expand the current understanding of the predictors and impact of PVT, contributing to its prevention and ultimately improving long-term outcomes in children undergoing LT.

## Supplementary Material


