# Supplementary material for: Portal Vein Thrombosis in Pediatric Liver Transplantation With Technical Variant Grafts: An International Multicenter Analysis of Risk and Prognostic Factors
Source: Transplantation. 2026 Jun 9;110(8):e1695–704. doi: 10.1097/TP.0000000000005777 (PMC13384361; doi:10.1097/TP.0000000000005777)

SDC, Material and Methods, Table S1. Clinically Relevant Variables Included in Least Absolute Shrinkage and Selection Operator (LASSO) Regression

**Variables**
Age [months]
Sex, male
Weight [kg]
PVT pretransplant
Biliary atresia
Kasai procedure
PELD score
Graft type
Graft weight [g]
GRWR
Cold ischemia time [h]
Warm ischemia time [h]
Operation duration [h]
Intraoperative RBC transfusions [100mL]
ABO-incompatibility

Abbreviations: GRWR, Graft-to-Recipient weight ratio; PELD, Pediatric End-Stage Liver Disease; PVT, Portal vein thrombosis, RBC, Red blood cell.

SDC, Results, Table S2. Baseline Characteristics of Recipients, Donors, and Grafts by Treatment Modality for PVT

Characteristics	Non-Surgical Treatment (n = 44)	Surgical Treatment (n = 43)	P-value
Age, months, median [IQR]	7.7 [5.3, 11.1]	8 [7, 12.5]	0.249
Sex, male, n (%)	20 (45.5)	18 (41.9)	0.903
Weight, kg, median [IQR]	7.1 [6.4, 8.6]	6.6 [5.5, 8.1]	0.318
Height, m, median [IQR]	0.7 [0.6, 0.7]	0.67 [0.6, 0.8]	0.880
PVT pretransplant, n (%)	1 (2.3)	3 (7)	0.592
Etiology of liver disease, n (%)			
Biliary atresia	37 (84.1)	36 (83.7)	1.000
Other cholestatic diseases	40 (90.9)	37 (86)	0.708
Metabolic diseases	1 (2.3)	2 (4.7)	0.984
Pediatric acute liver failure	2 (4.5)	3 (7	0.979
Tumor	0 (0)	0 (0)	NA
Kasai procedure, n (%)	26 (59.1)	29 (67.4)	0.558
PELD score, median [IQR]	19.4 [14.8, 25.3]	22 [15.1, 26.6]	0.547

Characteristics	Non-Surgical Treatment<br>(n = 44)	Surgical Treatment<br>(n = 43)	P-value
Cold ischemia time, min, median [IQR]	25 [20, 89.3]	120 [62.5, 222]	0.001
Warm ischemia time, min, median [IQR]	30 [30, 38.3]	39 [34.5, 44.5]	<0.001
Operation duration, h, median [IQR]	6.2 [5, 7.5]	7.5 [6.1, 9.5]	0.004
GRWR, median [IQR]	3.4 [2.7, 4.1]	3.6 [2.1, 4.4]	0.671
Graft weight, g, median [IQR]	245 [220, 280]	250.5 [195.5, 296.3]	0.837
Preoperative Bilirubin Levels, mg/dl, median [IQR]	173.4 [64, 275.2]	126.5 [47.9, 292.4]	0.735
Preoperative Platelet Count, µL, median [IQR]	151 [79, 226]	164. [91, 221]	0.660
Age donor, months, median [IQR]	32 [26.5, 36.5]	34 [27.5, 37.5]	0.723
Weight donor, kg, median [IQR]	21.8 [19.3, 23.5]	24.4 [21.2, 27.2]	0.014
Graft type, n (%)			

Characteristics	Non-Surgical Treatment (n = 44)	Surgical Treatment (n = 43)	P-value
Left lateral segment graft	39 (90.7)	37 (86.0)	0.737
Left lobe graft	2 (4.7)	3 (7.0)	1.000
Right lobe graft	1 (2.3)	1 (2.3)	1.000
Extended right lobe graft	0 (0)	0 (0)	
Donor type, n (%)			0.384
Deceased transplant	8 (17.2)	6 (14)	
Living donor transplant	36 (81.8)	37 (86.0)	
Reduced graft	4 (9.1)	7 (16.3)	0.493
Intraoperative RBC transfusions, 100mL, median [IQR]	3 [2, 6]	3.6 [2.8, 4.7]	0.459

Characteristics	Non-Surgical Treatment (n = 44)	Surgical Treatment (n = 43)	P-value
Portal vein flow after reperfusion, ml/100g/min, median [IQR]	490.43 [347.8, 737.9]	236.48 [139.4, 315.6]	0.025
Hepatic artery thrombosis, n (%)	1 (2.3)	6 (14)	0.108
Portal modulation, n (%)	6 (13.6)	8 (18.6)	0.735
Anticoagulation, n (%)	34 (77.3)	19 (44.2)	0.003
ABO-incompatibility, n (%)	8 (18.2)	8 (18.6)	
Failure to rescue, n (%)	0 (0.0)	2 (33.3)	

Abbreviations: GRWR, Graft-to-Recipient weight ratio; MELD, Model for End-Stage Liver Disease; PELD, Pediatric End-Stage Liver Disease; PVT, Portal vein thrombosis, RBC, Red blood cell.

SDC, Results, Table S3. Association of Ischemia Times with PVT (Adjusted for Donor Source and Graft Type)

Variable	Adjusted OR (95% CI)	P-value
Cold ischemia time [h]	1.12 (0.99–1.27)	0.066
Warm ischemia time [h]	0.93 (0.81–1.03)	0.211

SDC, Results, Table S4. Subgroup Analysis of Biliary Atresia: Baseline Characteristics of Recipient, Donor and Graft by Presence of PVT

**Characteristics**	**No PVT** (n=1694)	**PVT** (n=81)	**P-value**
Age, months, median [IQR]	8 [6, 13.5]	7.1 [6, 10]	0.085
Sex, male, n (%)	796 (47)	32 (39.5)	0.228
Weight, kg, median [IQR]	7.3 [6.3, 9.3]	7 [6, 7.7]	0.004
PVT pretransplant, n (%)	36 (2.1)	4 (4.9)	0.199
Kasai procedure, n (%)	1279 (77.8)	54 (73)	0.412
Portal vein flow after reperfusion, ml/100g/min, median [IQR]	519.99 [379.3, 750.6]	459.5 [261.1, 657.6]	0.074
PELD score, median [IQR]	17.3 [10.2, 24]	18 [12.9, 24.3]	0.290
MELD score, median [IQR]	13.5 [8, 23.3]	10 [9, 21]	0.908
GRWR, median [IQR]	3.4 [2.58, 4.06]	3.6 [2.72, 4.30]	0.177
Graft weight, g, median [IQR]	259 [220, 300]	250 [213.5, 288.8]	0.226
Graft type, n (%)			
Left lateral segment graft	1528 (92.5)	66 (90.4)	0.652

Left lobe graft	98 (5.9)	3 (4.1)	0.692
Right lobe graft	11 (0.7)	2 (2.7)	0.189
Extended right lobe graft	2 (0.1)	0 (0.0)	1.000
Donor type, n (%)			0.214
Deceased transplant	174 (10.3)	10 (12.4)	
Living donor transplant	1520 (89.7)	71 (87.7)	
Reduced graft	257 (16.0)	11 (15)	0.605
ABO-incompatibility, n (%)	256 (15.4)	15 (18.8)	0.520

Abbreviations: GRWR, Graft-to-Recipient weight ratio; MELD, Model for End-Stage Liver Disease; PELD, Pediatric End-Stage Liver Disease; PVT, Portal vein thrombosis, RBC, Red blood cell.

SDC, Results, Table S5. Multivariable Analysis of PVT Risk Factors in Biliary Atresia Patients (Firth Regression)

**Variable**	**OR (95% CI)**	**P-value**
Age [months]	1.00 (0.98–1.01)	0.759
Weight [kg]	1.01 (0.97–1.05)	0.708
Kasai procedure	0.67 (0.39–1.15)	0.159
Portal modulation	3.29 (1.76–6.17)	0.001
Portal vein reconstruction	2.96 (1.70–5.14)	<0.001

SDC, Results, Table S6. Comparison of different Cox models for PVT Effects on Patient and Graft Survival in Biliary Atresia

	Patient Survival			Graft Survival		
**Model**	**HR**	**95% CI**	**P-value**	**HR**	**95% CI**	**P-value**
Univariate	2.23	1.07, 4.63	0.031	10.16	5.09, 20.3	<0.001
Minimally adjusted	2.20	1.01, 4.78	0.046	11.86	5.74, 24.5	<0.001
With centre cluster	2.20	0.86, 5.62	0.10	11.86	6.07, 23.1	<0.001

SDC, Results, Table S7. Multivariable Analysis of PVT Risk Factors in Small Children (Weight <10kg, Age <5 years)

**Characteristics**	**OR (95% CI)**	**P-value**
Age [months]	0.98 (0.92–1.05)	0.624
Weight [kg]	0.83 (0.66–1.04)	0.108
Cold ischemia time [h]	1.02 (0.91–1.15)	0.725
Operation duration [h]	1.01 (0.88–1.16)	0.891
Biliary atresia	1.81 (0.86–3.83)	0.119
Reduced Graft	0.69 (0.34–1.39)	0.298
Portal modulation	4.17 (1.86–9.35)	0.001
Portal vein reconstruction	2.11 (1.11–4.01)	0.023

FIGURE S1. Distribution of Cases across Centers

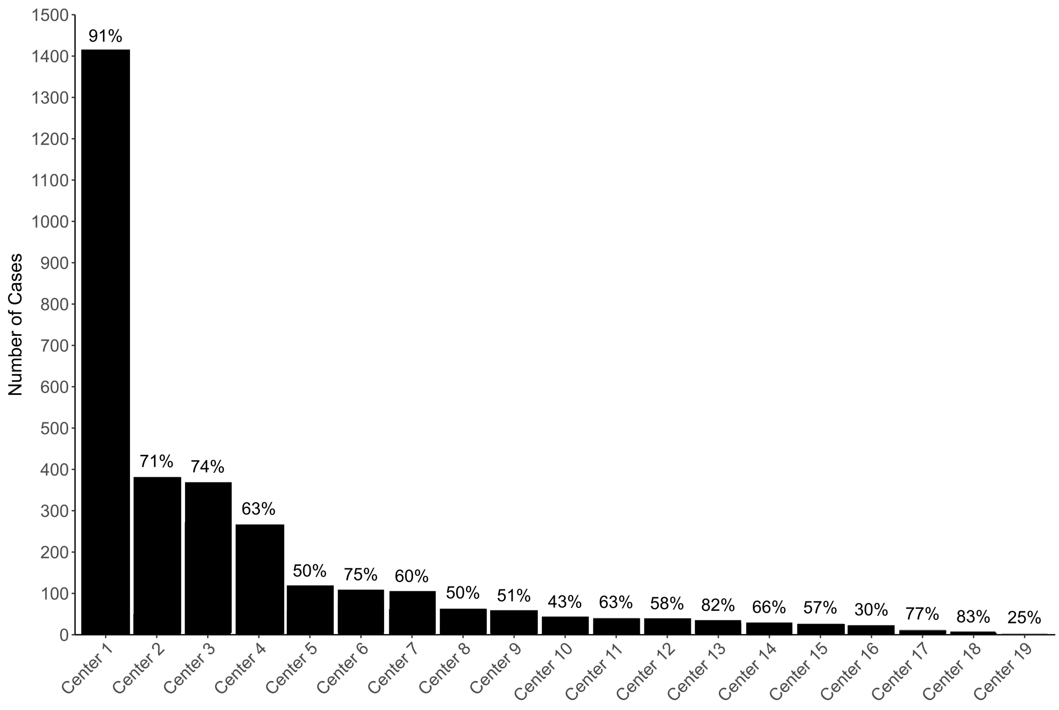

FIGURE S2. A) Cumulative incidence of Recanalisation, B) Recanalisation Following Surgical Management of PVT

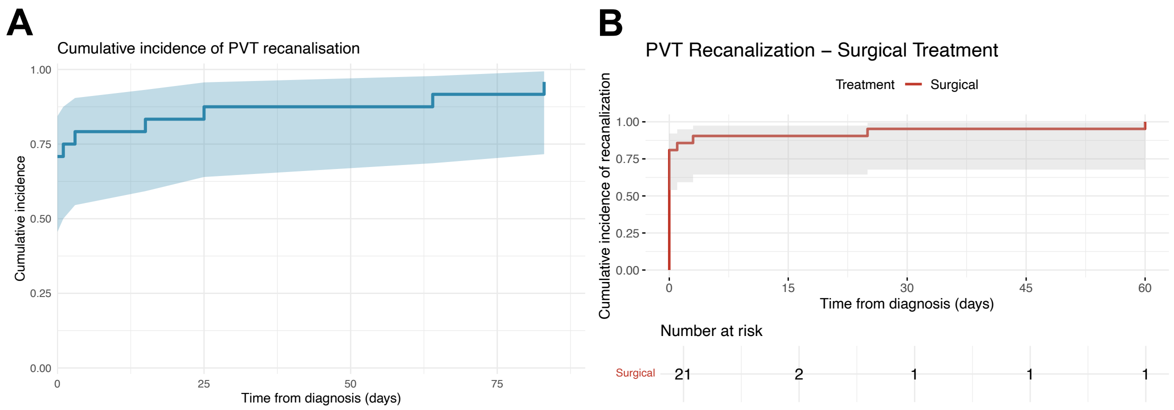

Supplement: Supplementary file 1 [file tpa-110-e1695-s001.pdf]
